# How Bacteria-Induced Apoptosis of Intestinal Epithelial Cells Contributes to Mucosal Inflammation

**DOI:** 10.4061/2010/574568

**Published:** 2010-07-06

**Authors:** Martin Hausmann

**Affiliations:** Division of Gastroenterology and Hepatology, Clinic of Gastroenterology and Hepatology, Department of Internal Medicine, University Hospital of Zürich, 8091 Zürich, Switzerland

## Abstract

The life cycle of an intestinal epithelial cell is terminated by apoptosis and/or cell shedding. Apoptotic deletion of epithelial cells from the intact intestinal mucosa is not accompanied by detectable inflammatory response or loss of barrier function. But increased permeability of the epithelial barrier and increased apoptotic rates of epithelial cells have been reported for patients suffering from inflammatory bowel disease. Microbiota can both induce or inhibit apoptosis of intestinal epithelial cells thus contribute to mucosal inflammation or support epithelial integrity respectively. Bacteria-mediated cytokine secretion and altered cell signalling are central to epithelial injury. Tumor necrosis factor (TNF) secreted after exposure to invasive bacteria induces both apoptosis and cell shedding. TNF is the major target gene of the transcription factor nuclear factor-kappa B with both pro- and anti-apoptotic effects. Autophagy promotes both cell survival and “autophagic” cell death. If autophagy is directed against microbes it is termed xenophagy. Inhibition of xenophagy has been shown to decrease cell survival. Endoplasmic reticulum (ER) stress causes misfolded proteins to accumulate in the ER lumen. It was suggested that ER stress and autophagy may interact within intestinal epithelial cells. Apoptosis in response to infection may be well proposed by the host to delete infected epithelial cells or could be a strategy of microbial pathogens to escape from exhausted cells to invade deeper mucosal layers for a prolonged bacterial colonization.

## 1. Introduction

The first boundary between self and nonself is constituted by intestinal epithelial cells. The intestinal mucosa forms a primary phalanx providing both barrier function and immediate effective recognition of bacterial products invading the mucosa. This is of great importance for the prevention of permanent and chronic inflammation as a reaction to the commensal intestinal flora and the multitude of antigens present in the intestinal lumen. Due to its enormous surface area, the barrier function of the intestinal mucosa may be as important as its function in nutrient absorption [1]. 

Intestinal epithelial cells are generated from stem cells at the lower third of the crypt and reach the intestinal lumen in 3–5 days. Apoptosis is initiated and cells finally lose anchorage and are shed into the lumen [2]. Apoptosis is a conserved genetic program for the removal of malfunctioning or potentially dangerous cells. Intestinal epithelial cells undergo apoptosis when they lose their contact with the extracellular matrix, a phenomenon termed “anoikis” [3]. This special form of cell death is likely to be the main mechanism terminating the physiological life cycle of intestinal epithelial cells, because they gradually lose cell anchorage on their march toward the intestinal lumen. Mechanisms terminating the short physiological life cycle of epithelial cells are supposed to have an impact on these essential functions and have influence on inflammatory reactions in the intestinal mucosa. A disturbance of the epithelial barrier is thought to be a major factor in the pathogenesis of inflammatory bowel disease. 

 This review focuses on recent significant research findings regarding mechanisms of bacteria-induced apoptosis, probiotic actions which promote host homeostasis, and the potential relevance of these mechanisms to mucosal inflammation and inflammatory bowel disease.

## 2. Cell Shedding, Sealing, and Healing

The life cycle of a nontransformed, intestinal epithelial cell is terminated by apoptosis or, if cells lose their contact with the matrix, by anoikis. The mechanism of cell shedding is important as abundant shedding must be achieved without loss of intestinal barrier function. An impermeable substance plugs epithelial discontinuities on the villus as a reaction to cell shedding, resulting in 3% of surface area having gaps near the villus tip [4]. Gaps contain no cellular material but are filled with a substance that maintains the epithelial barrier, despite discontinuities in the cellular layer. The source and composition of this “plugging” substance is unknown but could be secreted by neighboring epithelial cells, myofibroblasts, or the shed epithelial cell itself.

Physical wounding in human tissue culture models shows activation of myosin light chain kinase leading to contraction of an actin ring that, like a purse string, closes off the denuded region and restores barrier function [5]. Repair mechanisms after cell shedding in human histological sections have been characterized as similar to wound healing mechanisms of cultured epithelial monolayers [6]. During healing, myosin light chain kinase becomes phosphorylated at the edges of gaps in neighboring cells [6]. An actin ring at the apical pole of cells at the edge of the wound contracts like a purse-string contracture. For 45% of the shedding events, cytoplasmic extensions from the neighboring cells were shown to make a continuous structure beneath the shedding cell but tight junction activity of ZO-1 was only shown in 9% of the shedding events [4]. ZO-1 activity remains at the basal pole of gaps in a V-shaped formation. Thus it was mentioned that tight junctions from neighboring cells can only be used in a fraction of instances to help reseal the breach in the epithelial layer [4, 7]. Watson et al. considered that a number of shed cells do not have an apoptotic morphology or sufficient caspase activation [8]. Cells could be shed as largely intact or during early events in the apoptotic cascade. A forceful cell expulsion after loosening of contacts between neighboring intestinal cells and the basement membrane simultaneous with the secretion of an extracellular substance that fills the void left by the departing cell was mentioned [8]. Extracellular matrix molecules provide essential survival signals to epithelial cells via *β*1-integrins, focal adhesion kinase, and protein kinase B/Akt-1 pathways [9, 10]. Loss of cell-cell and cell-matrix contacts causes anoikis [3]. Preservation of cell-cell anchorage after *ex vivo* isolation of intestinal epithelial cells maintains survival. Src, PI3-K, and Akt, as well as the adherens junction protein *β*-catenin, are important elements of cell-cell anchorage-mediated antiapoptotic signals in intestinal epithelial cells [11]. Apoptotic deletion of epithelial cells from the intact intestinal mucosa is not accompanied by detectable inflammatory response and little, if any, disruptions of the intestinal epithelial barrier integrity are not the major route for bacterial translocation.

## 3. Implications for Disease

Disturbance of the epithelial barrier and epithelial transport processes is often discussed to be a major factor in the pathogenesis of intestinal inflammation and inflammatory bowel disease. Increased permeability of the epithelial barrier in ulcerative colitis was first reported in 1973 [11]. In a study by Hagiwara et al., epithelial apoptosis in mucosa from patients with ulcerative colitis was found to be considerably upregulated compared with control sigmoid colon [12]. An apoptotic rate of approximately 27.9% in patients ultimately requiring surgery for severe and/or steroid-refractory disease, 17.3% in patients receiving medication alone for more than 3 years, and 13.1% in patients with infectious colitis compared to 2.8% in the control colon. Increased permeability of the epithelial barrier in Crohn's disease was first reported in 1986 [13]. In a study by Zeissig et al., epithelial apoptosis in mucosa from patients with Crohn's disease was found to be considerably upregulated compared with control sigmoid colon [14]. An apoptotic rate of approximately 5.3% was obtained in the inflamed colon compared to 2.1% in the control colon. The apoptotic rate seems to be higher in ulcerative colitis than in Crohn's disease. Direct comparison of the apoptotic rate seems to be objectionable because of unequal experimental conditions. Abnormal gut permeability is characterized by measurably increased rates of apoptosis among epithelial cells. Intestinal bacteria are essential for the development of intestinal inflammation. A number of knockout models of inflammatory bowel disease confirm that bacteria are essential for the onset of inflammation [15–17].

## 4. Cytokine-Induced Apoptosis

### 4.1. TNF Increases Cell Shedding

Apoptosis can be induced by mucosal factors secreted by epithelial cells. Several studies have suggested that tumor necrosis factor (TNF) is central to epithelial injury. The epithelial cell lines T84, HT29, and Caco-2 secrete the proinflammatory cytokines interleukin (IL)-8, MCP-1, GM-CSF, and TNF after exposure to the gram-negative invasive bacteria *Salmonella dublin*, *Yersinia enterocolitica*, *Shigella dysenteriae,* and the gram-positive invasive bacterium *Listeria monocytogenes*. Expression of the same array of cytokines has also been shown in freshly isolated human colon epithelial cells [18, 19]. The noninvasive bacteria *Escherichia coli* DH5*α* and *Enterococcus faecium* had no significant effect on secretion [19, 20]. The cytokine TNF has been found to be elevated in inflammatory bowel disease where epithelial apoptosis is increased [21]. Accordingly, TNF induces both apoptosis and cell shedding when given to mice [22]. In intact intestinal mucosa, local barrier function was preserved during physiological cell shedding but was altered during TNF-induced loss of cells in mice. Approximately 20% of the TNF-evoked gaps were found to present no luminal permeability barrier [8]. TNF substantially increased gap formation and cell loss both individually and in sheets of up to 18 cells [23]. Extensive TNF-mediated apoptosis might be beyond the normal capacity to sustain gap impermeability. Supportive downregulation of epithelial apoptosis was found after TNF antibody treatment in patients with active Crohn's disease [14, 24] and in mouse models of colitis [24, 25]. As soon as microbes or their products have passed the epithelial barrier, TNF is also secreted by activated macrophages and T lymphocytes [26]. Apart from bacteria-induced apoptosis, dysregulated signals from the intestinal immune system are a major trigger of intestinal epithelial cell apoptosis. Intestinal mucosal biopsies from patients with active Crohn's disease contain increased numbers of TNF-secreting cells [27]. However, TNF initiates both antiapoptotic and proapoptotic signalling through the distinct receptors TNFR1 and 2 in a concentration [28, 29] and time- [30] dependent manner.

### 4.2. Probiotics Decrease TNF-Induced Cell Shedding

Several probiotics have been shown to prevent cytokine-induced apoptosis in epithelial cells. *Lactobacillus rhamnosus* GG (LGG), a bacterium used in the fabrication of yoghurt, prevents cytokine-induced apoptosis in mouse or human intestinal epithelial cells [31]. Studies on the relationship between LGG and both the murine colon epithelial cell line YAMC and the human colonic epithelial cell line HT-29 demonstrated that LGG inhibits TNF-induced apoptosis. LGG promotes the survival of cells through activation of the antiapoptotic Akt/protein kinase B in a PI3K-dependent manner and inhibition of the pro-apoptotic p38/mitogen-activated protein kinase activated by TNF, IL-1, or IFN*γ*. LGG produces soluble factors which can be recovered from bacterial culture without previous bacterial-intestinal cell contact [31]. LGG has been shown to reduce chemically induced intestinal epithelial apoptosis *in vitro* in IEC-6 and *ex vivo* in animal experiments [32]. Two novel proteins have been identified in LGG culture supernatant, p40 and p75; the first probiotic bacterial proteins demonstrated to promote intestinal epithelial homeostasis through specific signaling pathways [33]. These two factors are heat- and protease-sensitive [34]. Both p75 and p40 rescue TNF-induced epithelial damage, ulcerative lesions including disruption of epithelial integrity and epithelial apoptosis in cultured mouse colon explants. LGG has been shown to induce remission and prevent recurrence of inflammatory bowel disease in patients [35] as well as in animal models of colitis [36] but therapy in children with Crohn's disease showed no beneficial effect [37].

## 5. NF-*κ*B Deficiency Leads to Apoptosis in Intestinal Epithelial Cells

Bacteria display pathogen-associated molecular patterns recognized by Toll-like receptors (TLRs) at the surface of enterocytes required for the initiation of an inflammatory response. Intracellularly MyD88 transduces recognition signals for all bacteria. Downstream, inactive transcription factor nuclear factor-kappa B (NF-*κ*B) is complexed with inhibitory protein I*κ*B. NF-*κ*B is activated by dissociation of I*κ*B followed by ubiquitination and proteasomal degradation of the inhibitor. Dissociation of I*κ*B is induced through phosphorylation of inhibitor by I*κ*B kinase (IKK), a complex of two enzymatically active subunits IKK*α* and IKK*β* together with the regulatory subunit IKK*γ* (NEMO) [38]. NF-*κ*B is activated in intestinal epithelial cells of inflamed mucosa [39] and TNF is one of several prototypic NF-*κ*B target genes [40]. 

Contrary, physiological importance of TLR-signalling and NF-*κ*B-activation for intestinal homeostasis and maintenance of epithelial barrier has been shown in several recent studies. Inhibition of NF-*κ*B activation results in increased caspase activation and apoptosis [41]. TLR2-, TLR4-, and MyD88-deficient mice exhibited colonic bleeding [42]. Colons from MyD88-deficient mice showed severe and extensive denudation of the surface epithelium [42] but an increased number of proliferating epithelial cells. Dysregulated proliferation suggests the execution of a compensatory mechanism of intestinal epithelial cells as part of the physiological repair response to injury. Also, loss of TLR5 resulted in spontaneous colitis [43]. IKK*γ* (NEMO) deficiency in intestinal epithelial cells caused spontaneous colitis in mice associated with massive epithelial apoptosis, lost of barrier integrity, and translocation of commensal bacteria in the colon [44]. Analysis of proinflammatory gene expression showed upregulation of TNF in the colon [44]. A similar inflammation phenotype was observed in mice lacking both IKK*α* and IKK*β*. 

There are mixed results implicating NF-*κ*B in various models of apoptosis with both pro and antiapoptotic effects observed [41]. Downregulated expression of NF-*κ*B target genes with pro-survival functions in intestinal epithelial cells would be expected to exacerbate disease [38]. Completely absent NF-*κ*B-activation is followed by accelerated epithelial apoptosis and loss of epithelial barrier associated with enhanced exposure to bacteria. Inflammatory response is the total of the activation of both pro- and antiinflammatory signaling pathways in host cells.

## 6. More Probiotics, More Epithelial Cell Survival

Probiotics-mediated cytoprotection is the opposite of apoptosis. Uptake of probiotics is a therapeutic approach that regulates the homeostasis of intestinal epithelial cells by maintaining cell survival and enhancing barrier function. These nonpathogenic microorganisms have been used for the treatment of gastrointestinal diseases, including ulcerative colitis [45, 46] and Crohn's disease [34, 47]. Proposed mechanisms include bacterial interference, increased cytoprotection and decreased apoptosis of epithelial cells. Probiotic bacteria interact with three components of the gastrointestinal tract including competing bacteria, intestinal epithelial cells and mucosal immune cells [31].

### 6.1. Bacterial Interference: Microbe versus Microbe

Bacterial interference describes the condition in which a bacterial strain inhibits colonization by competing strains. The most plausible effect of bacterial interference is the antagonistic activity of probiotics against inflammation- and/or apoptosis-releasing pathogens. Probiotics prevent colonization by other pathogenic bacteria by the production of growth inhibitors including bacteriocins [48, 49] and microcins [50]. Probiotics were also shown to compete for adhesion sites on intestinal epithelial cells thereby replacing intestinal pathogens [51, 52]. Maintaining the integrity of intestinal epithelial cells by bacterial interference is especially relevant during defense against invasive pathogens. The probiotic *Escherichia coli* strain Nissle 1917 (EcN) is already in use for successful treatment of diseases of the digestive tract since several decades. EcN was demonstrated to inhibit invasion of *S. typhimurium*, *Y. enterocolitica*, *S. flexneri*, *L pneumophila* and *Listeria monocytogenes in vitro* [53]. The antiapoptotic effects found were due to soluble factors of EcN. Genomic islands encoding putative fitness factors were suggested as a genetic basis for the dominance of EcN upon other strains [54]. Fitness factors such as different iron uptake systems, adhesins, proteases [55], and the production of microcins [50] may support survival and successful colonization of EcN in the human gut and most likely contribute to its probiotic character. The anti-invasive mechanism of EcN is not dependent on direct physical contact to both epithelial cells and invasive bacteria. By separating EcN from the epithelial cell line with a porous membrane invasion is also effectively inhibited. The invasion systems used by bacterial strains blocked by EcN are diverse. An anti-invasive secreted component of EcN was postulated that acts on a central process or structure on the host [53]. A soluble or shed factor from EcN has been identified to induce *β*-defensin 2 in Caco-2, mediated through NF-*κ*B- and AP-1-dependent pathways. *β*-defensin 2 has a broad antibiotic spectrum against gram-negative and -positive bacteria. It may reinforce mucosal barrier by limiting bacterial adherence and invasion. In another study, flagellin has been shown to be a major stimulatory factor of EcN. The signal is transmitted by TLR5 [56].

### 6.2. Cytoprotective Effects Are Anti-Apoptotic Effects

Next to bacterial interference, more direct cytoprotective effects are described for soluble factors from probiotica. The probiotic mixture VSL#3 (*L. acidophilus*, *L. bulgaricus*, *L. Casei*, *L. plantarum*, *S. thermophilus*, *B. breve*, *B. infantis,* and *B. longum*) has been used as an effective treatment in ulcerative colitis patients [57]. The therapeutic effect of VSL#3 is reflected by increased cytoprotection of epithelial cells. 

An early event that occurs immediately after stimulation with VSL#3-conditioned medium *in vitro* is the inhibition of the proteasome [58]. Increase in ubiquitination is restricted to few proteins suggesting that particular features of the proteasome function are specifically inhibited. One of the proteins protected from proteasomal degradation is I*κ*B*α*. VSL#3-conditioned medium inhibits NF-*κ*B activation by stabilization of I*κ*B*α* [58]. Diminished NF-*κ*B activation inhibits endogenous immune response genes normally activated by NF-*κ*B, such as MCP-1. VSL#3-conditioned medium confers a degree of protection against oxidant injury through its ability to induce the expression of heat shock proteins (hsps) [58]. Induction of hsps occurs as a late event 12 hours after stimulation with VSL#3. Cytoprotective effects of hsps on epithelial cells are well characterized [59–61]. Hsp25 and 72 are induced by VSL#3 [58, 62]. Hsp25 preserves cytoskeletal and tight junction functions by binding to actin filaments and stabilizing F actin [63]. Hsp72 preserves critical cellular proteins and has been shown to protect epithelial cell lines against oxidant injury [64] and injury by NH_2_Cl [60].

## 7. Xenophagy and ER Stress in Intestinal Epithelial Cells: Novel Links toApoptosis-Related Disease Mechanisms?

Autophagy is a lysosomal degradation pathway that is essential for both survival and homeostasis [65]. Autophagy constitutes a stress adaptation pathway that promotes cell survival but is also considered to initiate a form of programmed “autophagic” cell death [65]. Autophagy is rapidly emerging as an important component of the innate immune response. Mutations in genes affecting the formation of autophagosomes [66, 67] or mutations in genes affecting lysosomal clearance of autophagosomes [68] are associated with increased frequency of apoptosis (reviewed in [65]). If autophagy is directed against invading microbes, it is termed xenophagy [69]. Xenophagy may have evolved as one of the first eukaryotic cell-autonomous defense mechanisms [70, 71]. 

Physiological states that increase the demand for protein folding, or stimuli that disrupt the reactions by which protein fold, create an imbalance between the protein-folding load and the capacity of the endoplasmic reticulum (ER), causing unfolded or misfolded proteins to accumulate in the ER lumen—a condition referred to as ER stress [72]. It was suggested that ER stress mechanisms and autophagic pathways may interact within intestinal epithelial cells *in vivo* and may be synergistically related to intestinal inflammation [73, 74].

### 7.1. Xenophagy

Autophagy directed against intracellular microbes or their products is termed xenophagy [69]. Yet most publications are focused on monocytes [75], macrophages [76, 77], fibroblasts [78, 79], and epithelial cells of nonintestinal origin [80] showing xenophagy-mediated killing of bacteria. Survival might be the benefit for a cell if pathogens are eliminated by xenophagy. Pathogens not eliminated by xenophagy might initiate apoptosis, sometimes accompanied by excessive inflammation.

During the last 3 years, xenophagy has also been investigated in intestinal epithelial cells. A number of single nucleotide polymorphisms (SNPs) showed clear genetic association with susceptibility to Crohn's disease [81, 82]. The strong genetic associations between Crohn's disease and both the autophagy-stimulatory immunity-related GTPase, IRGM1, and the autophagy execution gene, ATG16L, suggest a potential role for autophagy deregulation in the pathogenesis of Crohn's disease [65]. Interestingly, autophagy genes ATG16L1 and IRGM appear to be more specific for Crohn's disease [83] and IRGM risk alleles may predispose even more specifically to the ileal form of Crohn's disease [84, 85]. Deficiency in ATG16L1 had no effect on the overall morphology of the ileum or colon in mice [86] but abnormalities were identified in Paneth cells. Paneth cells are ileal epithelial cells known to secrete antimicrobial peptides and lysozyme. A lack of lysozyme was determined in the mucus of ATG16L1-deficient mice together with abnormal Paneth cell morphology. No evidence has been found for increased epithelial cell death or proliferation. Deletion of **A** **t** **g**5 in the intestinal epithelium in Atg5flox/floxvillin-Cre mice led to Paneth cell and granule abnormalities similar to those observed in Atg16L1 mice [86], while other epithelial cells appeared normal. This indicates that, within the intestinal epithelium, Paneth cells have a unique sensitivity to autophagy gene disruption. Mucosa from patients homozygous for the *ATG16L1* Crohn's disease risk allele contained Paneth cells with similar alterations to those in ATG16L1 deficient mice including cells with disorganized or diminished granules or cells exhibiting altered cytoplasmic lysozyme staining. Within the intestinal epithelium, the dramatic effect of autophagy protein deficiency on Paneth cells indicates that autophagy can contribute to disease pathogenesis via a highly specific role within a single cell lineage. However, involvement of enterocytes has not been determined so far.

In contrast, xenophagy can indeed act as a cellular defense pathway against secreted bacterial toxins in an apoptosis-dependent manner. Xenophagy targets the noninvasive intestinal pathogen Vibrio cholerae. Intestinal epithelial cells are the main target for the exotoxin Vibrio cholerae cytolysin (VCC). VCC causes extensive vacuolation in epithelial cells and autophagosome formation [87]. In several cell lines, including human intestinal CaCo-2 cells and C2BBe1, VCC-induced vacuoles colocalized with LC3, a clear sign of autophagosomal response. In contrast, LC3 remained cytosolic upon VCC treatment in Atg5−/− mouse embryonic fibroblasts. Autophagy is required to improve cell survival upon VCC intoxication. Both an inhibitor of autophagy and an inhibitor of autophagosome formation have been shown to produce a decrease in cell survival in CaCo-2 cells [87]. 

Xenophagy deficiency associated with a decrease in cell survival might contribute to a dysregulated immune response to commensal intestinal bacteria, altered mucosal barrier function, and defects in bacterial clearance [88, 89]. It will be essential to further determine whether increased epithelial apoptosis is a pathway induced as a part of xenophagy in human primary cells with different bacterial strains.

### 7.2. ER Stress

The cell has evolved highly specific signaling pathways, the unfolded protein response (UPR), to deal with ER stress conditions [90]. If UPR is not sufficient, the cell enters apoptosis [91]. Three UPR pathways have been described. The PERK/eIF2*α* pathway is mediated by a transcription factor called CHOP/GADD153. CHOP forms heterodimers with C/EBP*α* to induce transcription of Bim in various cell types including kidney epithelial cells, mouse embryonic fibroblasts, thymocytes, a cancer cell line, and a myeloid progenitor *cell line* [92]. Bim is a member of the pro-apoptotic protein family and counteract with the anti-apoptotic protein Bcl-2 to promote apoptosis. Stress stimuli trigger Bim translocation to the mitochondrial surface where it neutralize the anti-apoptotic action of Bcl-2 and induce the release of cytochrome c which finally initiates apoptosis. Bim protein is dephosphorylated by protein phosphatase 2A during ER stress and is thereby rendered resistant to ubiquitin/proteasome-mediated degradation [92]. Other UPR transducers are the activating transcription factor 6 (ATF6) and the inositol-requiring enzyme 1 (IRE1) [91, 93]. Two homologous genes encode IRE1, for which there is a ubiquitously expressed alpha form and a constitutively expressed beta form restricted to the intestinal epithelium [94, 95]. IRE1*β* has been shown to promote resistance to DSS-induced colitis [96]. The glucose regulated protein-78 (GRP78/BiP) is a central regulator of the concerted cellular response of the three UPR transducers IRE1*α*, PERK, and ATF6. Several factors are required for optimum protein folding, including ATP, Ca^2+^, and an oxidizing environment to allow disulphide-bond formation [97]. As a consequence of this specialist environment, the ER is highly sensitive to stresses that perturb cellular energy levels, the redox state, or Ca^2+^ concentration. Such stresses reduce the protein folding capacity of the ER, which results in the accumulation and aggregation of unfolded proteins [93]. 

Increased expression level of the ER stress chaperone grp-78 in colonic epithelial cells is associated with acute and chronic inflammation in inflammatory bowel disease. Purified human intestinal epithelial cells from patients with Crohn's disease and ulcerative colitis revealed increased expression levels of grp-78 comparing noninflamed and inflamed tissues [98]. As sigmoid diverticulitis patients revealed similar expression levels, grp-78-mediated ER stress may be part of the inflammatory processes rather than a specific feature of inflammatory bowel disease. The regulatory cytokine IL-10 was shown to partly inhibit ER stress responses in intestinal epithelial cells in mice after colonization with *E. faecalis* [98, 99]. TLRs play an important role in the recognition of microbial molecular patterns. It has been shown that ER stress-mediated epithelial apoptosis is associated with the expression of TLRs. A majority of regulated proteins from a comparison between wild-type mice and TLR2-deficient mice have been shown to be involved in energy metabolism and stress responses [100]. Under noninflamed and inflamed conditions, a lack of TLR-dependent signals leads to the induction of ER stress-associated mechanisms in primary intestinal epithelial cells in the large intestine of TLR2 knockout mice.

A number of pathways have been described that induce apoptosis by ER stress (reviewed elsewhere [91]). Indeed microbes can also directly affect ER stress pathways. Trierixin, a triene-ansamycin group compound from a culture broth of *Streptomyces sp.,* has been shown to directly inhibit transcription factor X-box-binding protein 1 (XBP1)-activation [101]. XBP1 deficiency increases susceptibility to colitis in the DSS mouse model [73]. XBP1 level has been shown to be increased in inflamed and noninflamed colonic biopsies from patients with Crohn's disease and ulcerative colitis. SNPs within the XPB1 gene region are associated with inflammatory bowel disease. Similar to deficiency in ATG16L1 Xbp1−/− intestine was completely devoid of Paneth cells [73]. Increased apoptosis was determined in Paneth cells from Xbp1 deficient mice but no evidence has been found for increased epithelial cell death or proliferation.

## 8. Conclusion

Induction of apoptosis in pathogenesis remains difficult to understand. Breakdown of the epithelial barrier play a part in the disruption of epithelial defenses and further accelerates mucosal inflammation. The potency of intestinal epithelial cells to identify bacteria through a set of pattern recognition receptors and adequately respond to infection, ultimately with apoptosis of the epithelial cell itself, is not a nonspecific phenomenon that only reflects severity of inflammation. Increased frequency of apoptosis is not synonymic with exceeded histiocytic phagocytic capacity. If human colon epithelial cells undergo exaggerated apoptosis in response to infection with pathogenic bacteria, this may be well proposed by the host itself to delete infected and damaged epithelial cells [102]. Execution of apoptosis is initiated by a proinflammatory program and induced either by immune competent cells or epithelial cells. On the other hand, apoptosis could be a strategy of microbial pathogens to escape from the infected and exhausted host cell to invade deeper mucosal layers for a prolonged bacterial colonization. Anti-apoptotic effects of probiotics may be due to blockade of NF-*κ*B activity and hsp induction.

Balance models are typically discussed to explain the onset of inflammation in the mucosal immune system. Equilibration indeed could be the topic for the mechanisms mentioned above. It will be important to evaluate the complex balance between pro- and anti-apoptotic signals generated by activated transcription factors and cytokines. Impact of xenophagy and ER stress on the molecular network of defence should be analysed in primary intestinal epithelial cells in the future.

## Abbreviations

ATG: Autophagy execution gene
ER: Endoplasmic reticulum
GRP78: Glucose regulated protein-78
IKK: I*κ*B kinase
IRGM: Immunity-related GTPase
IL: Interleukin
LGG: *Lactobacillus rhamnosus* GG
NF-*κ*B: Nuclear factor-kappaB
SNPs: Single nucleotide polymorphisms
TLRs: Toll-like receptors
TNF: Tumor necrosis factor
UPR: Unfolded protein response
VCC: Vibrio cholerae cytolysin
XBP1: X-box-binding protein 1.
